# Central and peripheral arterial stiffness responses to uninterrupted prolonged sitting combined with a high-fat meal: a randomized controlled crossover trial

**DOI:** 10.1038/s41440-021-00708-z

**Published:** 2021-08-02

**Authors:** Simon Fryer, Keeron Stone, Craig Paterson, Meghan Brown, James Faulkner, Danielle Lambrick, Daniel Credeur, Gabriel Zieff, Aitor Martínez Aguirre-Betolaza, Lee Stoner

**Affiliations:** 1 School of Sport and Exercise, University of Gloucestershire, Gloucestershire, UK; 2 School of Health Sciences, Birmingham City University, Birmingham, UK; 3 Department of Sport and Exercise, University of Winchester, Hampshire, UK; 4 School of Health Sciences, University of Southampton, Hampshire, UK; 5 Department of Biology, Ave Maria University, Ave Maria, FL USA; 6 Department of Sport and Exercise, University of North Carolina, Chapel Hill, NC USA; 7 Department of Physical Education and Sport, Faculty of Education and Sport, University of the Basque Country (UPV/EHU), Basque Country, Spain

**Keywords:** Vascular function, Sedentary behavior, Dietary behavior, Hypertension

## Abstract

Independently, prolonged uninterrupted sitting and the consumption of a meal high in saturated fats acutely disrupt normal cardiovascular function. Currently, the acute effects of these behaviors performed in combination on arterial stiffness, a marker of cardiovascular health, are unknown. This study sought to determine the effect of consuming a high-fat meal (Δ = 51 g fat) in conjunction with prolonged uninterrupted sitting (180 min) on measures of central and peripheral arterial stiffness. Using a randomized crossover design, 13 young healthy males consumed a high-fat (61 g) or low-fat (10 g) meal before 180 min of uninterrupted sitting. Carotid-femoral (cf) and femoral-ankle (fa) pulse wave velocity (PWV), aortic-femoral stiffness gradient (af-SG), superficial femoral PWV beta (β), and oscillometric pulse wave analysis outcomes were assessed pre and post sitting. cfPWV increased significantly more following the high-fat (mean difference [MD] = 0.59 m·s^−1^) meal than following the low-fat (MD = 0.2 m·s^−1^) meal, with no change in faPWV in either condition. The af-SG significantly decreased (worsened) (η_p_^2^ = 0.569) over time in the high- and low-fat conditions (ratio = 0.1 and 0.1, respectively). Superficial femoral PWV_β_ significantly increased over time in the high- and low-fat conditions (η_p_^2^ = 0.321; 0.8 and 0.4 m·s^−1^, respectively). Triglycerides increased over time in the high-fat trial only (η_p_^2^ = 0.761). There were no significant changes in blood pressure. Consuming a high-fat meal prior to 180 min of uninterrupted sitting augments markers of cardiovascular disease risk more than consuming a low-fat meal prior to sitting.

## Introduction

Epidemiological data suggest that prolonged sitting time [1] and the consumption of meals high in saturated fats [2] are behaviors that independently increase cardiovascular disease (CVD) risk [3]. There also appears to be a tendency for individuals to perform these two behaviors in combination [4], potentially further increasing CVD risk [5]. Fortunately, these risk factors are suggested to be two of the most modifiable behaviors for CVD risk reduction [3]. However, there is a paucity of research targeted at understanding the physiological mechanisms associated with prolonged sitting in combination with the consumption of meals of different nutritional compositions. In the only study to date, Cho et al. [6] reported that following 240 min of prolonged sitting after a high-fat meal, popliteal blood flow and shear rate were reduced, and this was subsequently improved with sitting interruptions in the form of stair climbing [6]. While these findings support the implementation of physical activity interventions to attenuate the deleterious effects of sitting, the study had no low-fat “control group.” Consequently, it is unclear whether the impaired arterial health was a result of predominantly the high-fat meal, the prolonged sitting period, or a consequence of performing these behaviors in combination. Currently, it is not known what the effects of prolonged uninterrupted sitting with and without a high-fat meal are on cardiovascular function.

Independently, the cardiovascular consequences of prolonged uninterrupted sitting and the consumption of a meal high in saturated fat have been well characterized [7, 8]. Several studies provide evidence to support the association between postprandially elevated triglyceride concentrations and endothelial dysfunction. For example, Vogel et al. [9] reported that compared to the consumption of a low-fat meal (0 g), the consumption of a one-off meal that is high in triglyceride-rich lipoproteins (50 g) reduces endothelial function by 11% between 2 and 4 h after the meal. This triglyceride-induced endothelial dysfunction is likely due to a combination of both acute morphological and cellular changes. Independently, endothelial function, assessed using flow-mediated dilation, has also been shown to be reduced (2.12%) by periods of prolonged uninterrupted sitting [7]. Interestingly, prolonged sitting has also been shown to negatively impact systemic arterial health with changes in both central and peripheral pulse wave velocity (PWV) [10]. PWV is considered to be the gold standard assessment for arterial stiffness [11, 12], is a proxy for endothelial function [13], and can be used to reliably and accurately assess both central and peripheral vascular function. In addition to regional PWV measures, the interaction between central and peripheral vasculature, termed the “stiffness gradient,” has been employed as a novel way to investigate vascular function [14]. Indeed, a reduction in the stiffness gradient has been previously linked to target end organ damage [15] and likely decreases with prolonged uninterrupted sitting or the consumption of a single high-fat meal.

As such, this study sought to determine the effect of consuming a high-fat meal (Δ50 g) in conjunction with a period of prolonged uninterrupted sitting (180 min) on central and peripheral arterial stiffness. Specifically, the aims were to determine the effects of a high-fat meal vs. those of a low-fat meal in conjunction with prolonged sitting on (1) central and peripheral PWV, (2) the novel aortic-femoral stiffness gradient (af-SG), and (3) local femoral artery blood flow and stiffness measures.

## Methodology

This study is reported in accordance with the Consolidated Standards of Reporting Trials guidelines [16].

### Participants

Thirteen healthy nonsmoking male participants were recruited. Participant characteristics were as follows: age 22.3 ± 2.2 years, height 178 ± 6 cm, and mass 76.7 ± 9.2 kg. Self-reported data suggested that all participants were physically active, exercising 3.8 ± 2.1 times per week for 2.2 ± 1.8 h per session. Written informed consent and institutional ethical approval were obtained.

### Experimental protocol

Participants visited the laboratory on three separate occasions. During the first visit, participants were familiarized with all equipment and experimental procedures. Height, body mass, physical activity status, and food allergies or intolerances were determined. The order of the following two experimental visits were randomized (using https://www.randomizer.org/), and the visits were separated by at least 48 h but no more than 10 days. Participants were blinded to the meal type until the start of their first trial visit. Each visit began at 8:30 a.m. following an overnight fast. Participants abstained from consuming alcohol and engaging in strenuous exercise for a 24-h period prior to each visit. Prior to the first visit, the participants recorded their evening meal and were asked to repeat this meal before their subsequent assessment. This study required participants to engage in 180 min of sitting, as this has been shown to impair PWV [10], and for comparative purposes, it is currently the most frequently reported time period used for assessing vascular function [7]. During the 180-min sitting period, participants were asked not to partake in boisterous activities but were allowed to watch a nonstimulatory television documentary.

At each experimental visit, participants quietly lay supine on a test bed for 20 min while being fitted with an oscillometric blood pressure cuff (SphygmoCor Xcel, AtCor Medical, Sydney, Australia) over their left upper arm, and thigh and ankle cuffs were placed on the left side of the body to determine central and peripheral blood pressures and carotid-femoral (cf) PWV (cfPWV) and femoral-ankle (fa) PWV (faPWV), respectively. A continuous-wave near-infrared spectroscopy (NIRS) (Artinis Medical Systems, BV Zetten, the Netherlands) device was placed over the muscle belly of the dominant gastrocnemius to determine changes in blood volume (blood pooling). To ensure that the NIRS unit was on the same portion of the gastrocnemius for each trial, the skin was marked with an indelible pen, distances from anatomical locations were recorded, and photos were taken.

Following 20 min of supine rest, a Doppler ultrasound (Terason T3300, Burlington, MA, USA) with a linear array probe (15–4 MHz) collected 3 × 10 s videos of the superficial femoral artery (SFA) on the left side of the body 2 cm distal to the bifurcation. Oscillometric pressure waveforms were assessed using brachial cuff inflation. Subsequently, the SphygmoCor Xcel was used to determine cfPWV followed by faPWV; the af-SG was subsequently calculated offline. The participant was then manually moved into a seated position using an electronic three-way tilt table (Plinth 2000, Plinth Medical, Suffolk, UK), and baseline triglyceride concentration was assessed. Calf circumference was determined on the dominant gastrocnemius by measuring the area of greatest girth, which was subsequently marked, and the distance from anatomical landmarks was recorded for subsequent trials. The participant was then given 10 min to eat their breakfast. All participants finished their allocated meals. Participants were asked not to move their legs during the 180-min period of sitting, as leg fidgeting has previously been shown to improve lower-limb vascular function [17], and our study aimed to minimize potential cofounding variables between trials as much as possible. Participants were able to urinate in situ if required. No participants needed to empty their bowels during any visits. Blood samples for determination of triglycerides were taken at 30, 60, 120, and 170 min of sitting. Following 180 min of sitting, all assessments were repeated.

## Experimental procedures

### Meal type

In accordance with previous research [9], we used a McDonald’s Corporation breakfast meal, which included a double sausage and egg McMuffin, two hash browns, and hot chocolate with added double cream (1066 kcal, 4.5 MJ, 61 g fat [of which 20 g was saturated fat], 86 g carbohydrates, 40 g protein, and 5 g salt). The low-fat meal consisted of two large English crumpets (Kingsmill, Allied Bakeries, London, UK), each with 10 g of low-fat spread (Tesco PLC), 5 g of marmite, and a 200 mL skimmed milk beverage with 22 g of unflavored whey protein powder (MYPROTEIN) (601 kcal, 2.5 MJ, 10 g fat [of which 3 g was saturated fat], 86 g carbohydrate, 40 g protein, 5 g salt). A 51-g difference in fat was used, as 50 g has previously been shown to independently cause endothelial dysfunction in healthy individuals [9].

### Regional PWV

The SphygmoCor XCEL device was used to assess cfPWV and faPWV. PWV is calculated by dividing pulse transit time (PTT) by arterial path length (*D*). For cfPWV, the tonometer was placed on the left carotid artery, and the oscillometric cuff was placed on the upper left thigh, following recommended manufacturer guidelines [18]. The cf *D* was estimated by measuring the linear distance from the suprasternal notch to the top of the leg cuff and subtracting the distance from the suprasternal notch to the carotid artery. For faPWV, the tonometer was placed on the SFA, while the ankle cuff (SC10, Hokanson) was positioned at the malleolus. Fa *D* was estimated by measuring the linear distance between these two points. Fa PTT was corrected prior to the calculation of PWV as previously described [19]. A PWV stiffness gradient was calculated as cfPWV/faPWV [15].

### Local PWV and blood flow

Local measures of femoral artery PWV_*ß*_ and blood flow provide additional mechanistic information that complements regional arterial stiffness measures when determining the effects of prolonged sitting on arterial function [20]. Three 10-s videos of the ultrasound readings were recorded using external video-capturing software (LiteCam HD, Englewood Cliffs, NJ, USA). During each 10-s video capture, participants were instructed to hold their breath (without taking a large breath) throughout the measurement. The video clips were analyzed offline using automated edge-detecting software (FMD Studio, Quipu, Italy) by a trained operator blinded to the condition. Custom written Excel Visual Basic code was used to fit peaks and troughs to the diameter waveforms to calculate diastolic, systolic, and mean diameters. Blood flow was calculated from continuous diameter and mean blood velocity recordings using the following equation: 3.14 × (diameter/2)^2^ × mean blood velocity × 60. A local, single-point measure of PWV was calculated using the PWV_*ß*_ equation, previously described in detail by Fryer et al. [20].

### Pulse wave analysis (PWA)

SphygmoCor Xcel was used to conduct PWA. Oscillometric pressure waveforms are assessed during brachial cuff inflation, and a corresponding aortic waveform is generated using a validated transfer function [21]. From subdiastolic recordings, central systolic blood pressure, central diastolic blood pressure, central pulse pressure, central augmentation pressure, augmentation index (AIx), AIx normalized to a heart rate of 75 bpm (AIx@75), forward aortic pressure, backward aortic pressure (Pb), and subendocardial viability ratio were derived.

### Blood sampling

Using a 1.6 mm lancet, all capillary blood samples were collected using a 32 μL lithium heparin capillary tube (Sarstedt Aktiengesellschaft & Co, Germany). Samples were extracted onto a Reflotron test strip (Hoffmann-La Roche Ltd) for determination of triglyceride concentration (mg · dL) using a Reflotron Plus (Hoffmann-La Roche Ltd).

### Near-infrared spectroscopy

A Portalite continuous-wave NIRS device was used to determine blood volume in the gastrocnemius as a measure of blood pooling before and after 180 min of uninterrupted sitting. Portalite permits the determination of oxyhemoglobin and deoxyhemoglobin, the sum of which is total hemoglobin (tHb). Changes in tHb using NIRS have previously been shown to be both valid and reliable for the assessment of lower-limb blood pooling during an orthostatic challenge [22].

### Sample size

Using the effect size of 0.36 derived from the main effect of time, i.e., change in cfPWV between pre and post 180 min of sitting [10], the maximum chance of a type 1 error set at 5% (i.e., very unlikely) and power set at 0.8, the approximate number of participants required according to G*Power, was 12. However, 13 participants were recruited to account for any data errors or participant attrition throughout the study.

### Statistical analysis

Statistical analyses were performed using Statistical Package for Social Sciences version 25 (IBM, Chicago, IL). According to the Shapiro–Wilk and Mauchly’s test of sphericity, all dependent variables were found to be normally distributed and spherical. In accordance with Fryer et al. [20], all PWV, PWA, and local blood flow measures were collected three times, and the average of the closest two values was reported. All data are reported as the mean, standard deviation (SD), mean difference (MD), and 95% confidence interval (CI) unless otherwise stated. For all two-way repeated-measures ANOVAs where a significant interaction of time × condition was found, separate one-way ANOVAs with post hoc Bonferroni were conducted on each condition. When a main effect was reported, paired samples *t*-tests with the MD and 95% CI were reported. Eta^2^ (η_p_^2^) was used as a measure of effect size for ANOVA, where 0.01, 0.06, and 0.14 represented a small, medium, and large effect, respectively [23]. As a measure of effect size for *t*-tests, Cohen’s *d* was used, where 0.2, 0.5, and 0.8 represented a small, medium, and large effect, respectively [23]. α was set at *p* < 0.05 (two tailed).

## Results

### Triglyceride concentration

**Fig. 1:**
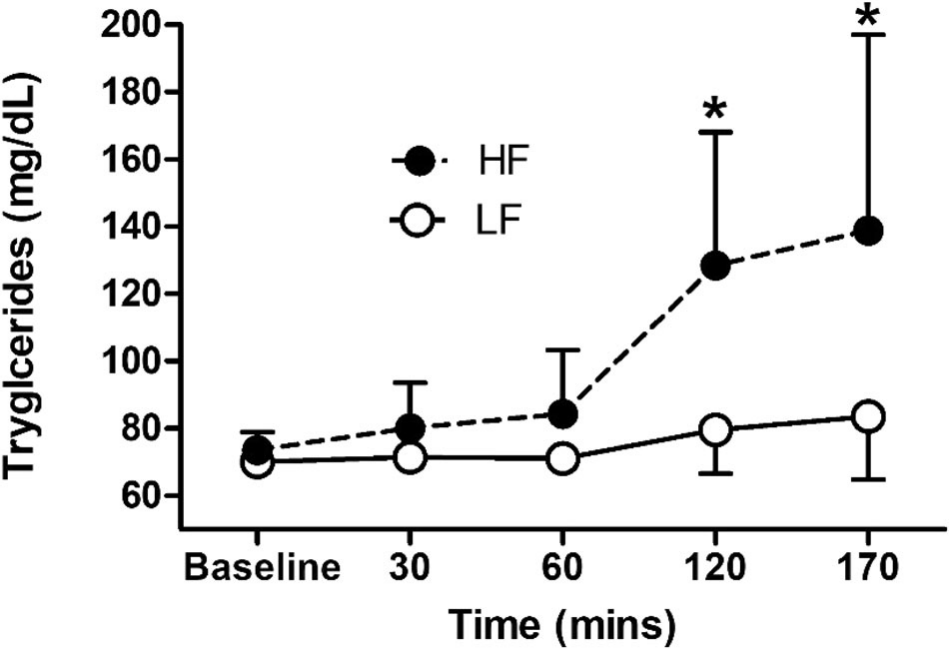
Mean ± SD triglyceride concentrations (mg · dL) during prolonged sitting following high and low-fat meal consumption. NB: mg · dL; HF high fat; LF low fat; asterisk (*) denotes significantly different from baseline

As shown in Fig. 1, there was a significant interaction for triglyceride concentration (η_p_^2^ = 0.615, *p* = 0.0001). Follow-up analyses revealed that the triglyceride concentration did not change over time in the low-fat condition. However, there was a significant main effect of time for the high-fat condition (η_p_^2^ = 0.761, *p* = 0.001), with the triglyceride concentration significantly increasing from pre to post 120 min (MD = 55, 95% CI = 18–91 mg/dL, Cohen’s *d* = 1.85) and 170 min (MD = 65, 95% CI = 11–120 mg/dL, Cohen’s *d* = 1.52).

### Calf circumference and blood pooling (tHb)

**Fig. 2:**
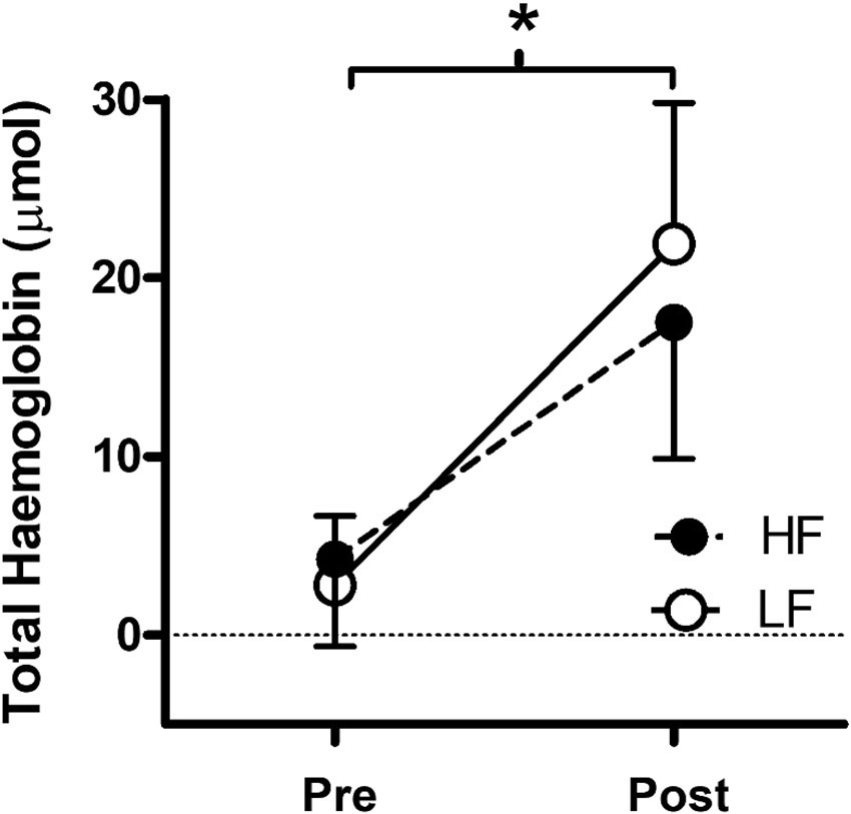
Mean ± SD total hemoglobin (μmol) in the gastrocnemius at 0 and 180 min of sitting following a low and high-fat meal. NB: asterisk (*) denotes significant (*p* < 0.05) main effect of time

There was no significant interaction (*p* = 0.089, η_p_^2^ = 0.378) for tHb. However, as seen in Fig. 2, there was a significant main effect of time (*p* < 0.001, η_p_^2^ = 0.877) but not condition (*p* = 0.492, η_p_^2^ = 0.061). tHb increased similarly in the high-fat (MD = 14.3, 95% CI = 8.4–20.3 µmol) and low-fat (MD = 19.1, 95% CI = 14.5–23.7 µmol) conditions. For calf circumference, there was no significant interaction and no main effect of group; however, there was a significant main effect of time (*p* < 0.001, η_p_^2^ = 0.928). Calf circumference increased similarly over time in both low-fat (MD = 1.42, 95% CI = 0.94–1.89 cm) and high-fat (MD = 1.66, 95% CI = 0.18–2.06 cm) conditions, indicating similar effects on blood pooling.

### Pulse wave velocity

As reported in Table 1, a significant interaction for cfPWV (*F*_(1,12)_ = 7.221, *p* = 0.02; η_p_^2^ = 0.376) was found. Follow-up analyses revealed no significant change in cfPWV over the 180-min period of sitting for the low-fat condition (post hoc MD = 0.14, 95% CI 0.05–0.34 m·s^−1^, Cohen’s *d* = 0.44), but a significant increase was found following the high-fat condition (post hoc MD = 0.59, 95% CI 0.29–0.89 m·s^−1^, Cohen’s *d* = 1.12). For faPWV, there was no significant interaction or main effect. For the af-SG, there was no significant interaction effect or group effect, but there was a significant main effect of time.

**Table 1:** Mean (±SD), interaction, and main effects for central and peripheral PWV pre (0 min) and post (180 min) prolonged sitting following a low- and high-fat meal

		cfPWV (m·s^−1^)	faPWV (m·s^−1^)	af-SG
Mean average (SD)				
Low fat	0 min	5.8 (0.7)	9.3 (1.1)	1.6 (0.2)
	180 min	6.0 (0.5)^b^	9.2 (1.1)	1.5 (0.2)^b^
High fat	0 min	5.5 (0.5)	9.1 (1.1)	1.6 (0.1)
	180 min	6.1 (0.6)^a,b^	9.1 (1.1)	1.5 (0.2)^b^
Interaction effect				
	*p*	0.020	0.689	0.154
	η_p_^2^	0.376	0.014	0.162
Time effect				
	*p*	0.001	0.538	0.002
	η_p_^2^	0.640	0.032	0.569
Condition effect				
	*p*	0.671	0.418	0.751
	η_p_^2^	0.016	0.055	0.009

*SD* standard deviation, *η*_p_^2^ partial Eta^2^, *p* significance, *cfPWV* carotid to femoral pulse wave velocity, *faPWV* femoral to ankle pulse wave velocity, *af-SG* aortic-femoral stiffness gradient

^a^Significant interaction effect

^b^Significant time effect

### Local femoral artery measures

As shown in Table 2, there were no significant interactions or main effects for blood flow, diameter, or shear rate at the femoral artery. However, there was a significant main effect of time for femoral artery diameter and PWV_*ß*_.

**Table 2:** Mean (±SD), interaction, and main effects for local superficial femoral artery measures pre (0 min) and post (180 min) prolonged sitting following a low- and high-fat meal

		Superficial femoral artery			
		Blood flow (mL·min)	Shear rate (S^−1^)	Avg. diameter (mm)	PWV_*ß*_ (m·s^−1^)
Mean average (SD)					
Low fat	0 min	290 (162)	87 (24)	6.6 (0.9)	5.9 (1.6)
	180 min	281 (158)	89 (15)	6.4 (0.9)	6.3 (1.1)
High fat	0 min	296 (117)	93 (32)	6.5 (0.9)	5.6 (0.7)
	180 min	264 (116)	100 (32)	6.3 (0.9)^a^	6.4 (0.9)^a^
Interaction effect					
	*p*	0.271	0.493	0.637	0.409
	η_p_^2^	0.132	0.044	0.019	0.063
Time effect					
	*p*	0.060	0.196	0.030	0.044
	η_p_^2^	0.340	0.147	0.336	0.321
Condition effect					
	*p*	0.834	0.091	0.599	0.400
	η_p_^2^	0.005	0.238	0.024	0.065

*SD* standard deviation, *η*_p_^2^ partial Eta^2^, *p* significance, *PWV*_*ß*_ pulse wave velocity beta

^a^Significant time effect

### Pulse wave analysis

As presented in Table 3, there was a significant interaction for AIx and AIx@75. Follow-up analyses found a nonsignificant change in AIx over the 180-min sitting period for the low-fat condition, but a significant decrease was found during the high-fat condition (MD = 6.8, 95% CI 4–9.6, Cohen’s *d* = 0.84). For HR, there was a significant main effect of time, with HR increasing over 180 min of sitting in both the low-fat (MD = 3, 95% CI 5–0.3 beats·min^−1^, Cohen’s *d* = 0.45) and high-fat (MD = 2, 95% CI 5–0.6 beats·min^−1^, Cohen’s *d* = 0.24) conditions. For all other PWA variables in Table 3, there were no significant interaction or main effects.

**Table 3:** Mean (±SD), interaction, and main effects for pulse wave analysis data pre (0 min) and post (180 min) prolonged sitting following a low- and high-fat meal

		Heart rate (beats · min)	SBP (mmHg)	MAP (mmHg)	cSBP (mmHg)	DBP (mmHg)	cPulse pressure (mmHg)	AIx (%)	AIx@75 (%)	Pf (mmHg)	Pb (mmHg)	SEVR (%)
Mean average (SD)												
Low fat	0 min	57 (6)	114 (10)	78 (6)	99 (8)	66 (5)	33 (6)	3 (9)	−6 (8)	25 (5)	14 (9)	171 (24)
	180 min	60 (6)	116 (9)	76 (8)	101 (6)	65 (3)	36 (8)	3 (7)	−4 (7)	27 (3)	12 (1)	160 (18)
High fat	0 min	59 (9)	118 (7)	78 (5)	102 (6)	65 (4)	37 (7)	7 (9)	−2 (9)	28 (4)	13 (2)	160 (23)
	180 min	61 (9)^b^	117 (10)	77 (6)	104 (7)	64 (7)	40 (8)	0.4 (7)^a,b^	−6 (6)^a^	28 (5)	11 (2)	155 (23)
Interaction effect												
	*p*	0.704	0.145	0.690	0.060	0.626	0.065	0.045	0.047	0.192	0.974	0.551
	η_p_^2^	0.012	0.168	0.014	0.321	0.020	0.255	0.295	0.290	0.138	<0.001	0.030
Time effect												
	*p*	0.029	0.687	0.446	0.626	0.567	0.803	0.010	0.319	0.153	0.416	0.126
	η_p_^2^	0.340	0.014	0.049	0.020	0.028	0.005	0.439	0.083	0.162	0.056	0.182
Condition effect												
	*p*	0.324	0.091	0.676	0.452	0.429	0.078	0.485	0.537	0.456	0.457	0.195
	η_p_^2^	0.081	0.219	0.015	0.048	0.053	0.237	0.041	0.033	0.008	0.047	0.136

*SD* standard deviation, *η*_p_^2^ partial Eta^2^, *p* significance, *mmHg* pressure, *AIx* augmentation index, *Pf* pressure forward, *Pb* pressure backward, *SEVR* subendocardial variability ratio, *SBP* systolic blood pressure, *MAP* mean arterial pressure, *cSBP* central systolic blood pressure, *cPP* central pulse pressure

^a^Significant interaction effect

^b^Significant time effect

## Discussion

The current study is the first to determine the effects of uninterrupted prolonged sitting in combination with a high-fat meal compared to uninterrupted sitting in combination with a low-fat meal. The main findings were as follows: (1) a high-fat meal immediately prior to 180 min of prolonged uninterrupted sitting caused a significant increase in cfPWV but not faPWV, and (2) 180 min of prolonged uninterrupted sitting caused a significant decrease in the af-SG (which is detrimental) and an increase in local (femoral artery) single-point PWV_*ß*_, irrespective of meal type.

### Limitations and strengths

While this is the first study to assess the effects of prolonged sitting with and without a high-fat meal on vascular function, to appropriately contextualize the findings, it is important to highlight the study’s limitations and strengths. First, as the fat content of the meal was not prescribed relative to body mass, some participants consumed relatively more than others. However, the consumption of 50 g of fat irrespective of participant body mass has previously been shown to reduce endothelial function in healthy adults [24], and given that the SD for participant body mass in the current study was small, any impact on our primary findings was likely to be minimal. Second, all participants were asked to ensure that their pretrial (night before) meals were consistent between visits. While it cannot be guaranteed that participants adhered to this instruction, they were reminded prior to the second session, and a food diary was completed to ensure compliance. Third, because the sample population was composed of habitually active males, the study’s findings cannot be generalized beyond this population. Fourth, as extraneous variables such as leg fidgeting [17] and short walking breaks [25] for the toilet may improve vascular functions such as blood flow, these activities were not allowed in the current study, and therefore, the ecological validity of the results is lower. Fifth, we did not assess habitual sitting time before either trial, and as a reduced step count prior to prolonged sitting can negatively impact vascular function [26], reductions in physical activity that were not accounted for may have affected our results. Compared to prior literature, a significant strength of the study is the within-participant design, whereby participants act as their own control. An additional strength of our study was the use of the af-SG as a novel marker of the integration of central and peripheral vasculature, which may provide additional information beyond standard PWV assessments [15].

### Comparison to the literature

cfPWV is considered the gold standard assessment of arterial stiffness [27] and is an independent predictor of CVD risk [28]. As such, our key finding that a high-fat meal followed by 180 min of uninterrupted sitting significantly increased cfPWV (MD = 0.6 m·s^−1^) more than a low-fat meal followed by sitting is an important one. Given that we found a significant interaction for triglyceride concentration (Fig. 1), with the high-fat intervention resulting in large increases between baseline and 120 min (MD = 55 mg/dL) and 170 min (MD = 65 mg/dL) post meal, it seems likely that the increase in cfPWV is caused by the addition of the high-fat meal. Furthermore, independent of sitting-induced changes in cfPWV, postprandial elevations in circulating triglycerides after a single meal containing 50 g of triglyceride-rich lipoproteins have been shown to impair endothelial function (flow-mediated dilation) by 11% [9]. It also seems likely that the high-fat meal contributed to the increased cfPWV, as the response in our study is greater than that previously reported in other nonmeal controlled studies [10]. Previously, Credeur et al. [10] reported that prolonged uninterrupted sitting alone (i.e., not dietary manipulation) produced a comparatively smaller increase in cfPWV (0.4 m·s^−1^) compared to that associated with the high-fat trial in the current study (0.6 m·s^−1^). As such, the findings of the current study suggest that the combined effect of consuming a high-fat meal prior to 180 min of prolonged uninterrupted sitting augments cfPWV, likely as a result of the combined behaviors worsening endothelial function. However, as the baseline measures in the current study were lower for the high-fat trial (0.27 m·s^−1^), further research should aim to understand whether a lower cfPWV is more sensitive to the deleterious effects caused by sitting and a high-fat meal. It should also be noted that as we did not assess step count prior to each trial and Boyle et al. [26] found that reducing step count before sitting worsened popliteal endothelial function following prolonged sitting, we cannot rule out that this factor did not play a part in the lower baseline high-fat cfPWV shown in Table 1.

In addition to the cfPWV assessment, we also determined regional (faPWV) and local (PWB_*ß*_) measures of arterial stiffness in the leg (Table 2). Interestingly, unlike cfPWV, there was no significant time effect for the regional stiffness measure faPWV, but there was a time effect for PWB_*ß*_. These differences in PWV measures may be caused by either the nonuniformity in stiffness across an artery [29], as endothelial function changes due to the increase in muscularity toward the periphery [30], or the fact that the local measure (PWB_*ß*_) may be more sensitive given that it is a direct measurement of endothelial function [13]. Previous research into the effects of prolonged uninterrupted sitting has found that 180 min of sitting caused a reduction in popliteal endothelial function [17], which was largely thought to be caused by a reduction in blood flow. While the current study found a reduction in SF artery blood flow over time, the reduction was not significantly different (*p* = 0.06), and the effect size was small (η_p_^2^ = 0.340). However, this reduction combined with the significant and large increase (η_p_^2^ = 0.877) in venous pooling and calf circumference may have in part caused the increase in local stiffness (PWB_*ß*_) at the SF artery.

Recently, a stiffness gradient has been presented as a novel marker of hemodynamic integration and CVD risk [14]. A stiffness gradient may provide unique insight into the acute consequences of prolonged sitting and high-fat meals on arterial health. Indeed, reductions in the central-to-peripheral arterial stiffness gradient can increase the transmission of pulsatile forces to the microcirculation, potentially leading to target organ damage [15, 31]. In the present study, the af-SG was significantly reduced (worsened) in response to 180 min of uninterrupted sitting, but this change was not augmented by the high-fat meal (Table 1). The consequences of this reduced gradient are likely to be limited in the relatively young population assessed in the present study, given that the stiffness gradient values were maintained above what is considered to be physiologically normal (i.e., values greater than 1.0). However, the central-to-peripheral arterial stiffness gradient decreases with age, and the reduction is accelerated by the presence of disease [14]. Accordingly, if the baseline af-SG is already below 1, then any further reduction in the stiffness gradient caused by prolonged sitting would likely heighten the risks of target organ damage. While there is currently no clinical af-SG threshold, future work should seek to identify whether the measure would be informative in elderly and at-risk populations.

Last, our study found that tHb significantly increased within the gastrocnemius following 180 min of sitting with no difference between meal types. This increase in lower-limb pooling likely plays a large part in explaining the significant decrease in both AIx and AIx@75 following 180 min of uninterrupted sitting. This finding complements that of Credeur et al. [10], who also reported a decrease in AIx over 180 min with an increase in calf circumference. While not investigated in the context of prolonged sitting, studies using orthostatic stressors (e.g., tilt test) have suggested that increased pooling leads to dampened pulse wave reflection [32], thus reducing AIx. To account for this, the reflected wave (Pb) is believed to mirror peripheral resistance to pressure waves descending from the aorta [21, 33]. However, as there were no changes in Pb or blood pressure (Table 3) in either the high-fat or low-fat trials, it is unlikely that peripheral resistance or changes in blood pressure contributed to the decreased Aix. However, given the effects that lower-limb pooling appears to have on AIx, its interpretation as a marker of systemic arterial stiffness during prolonged sitting trials may be questionable. Further research should determine the validity of using Aix and Aix@75 to assess systemic aortic stiffness in studies that encounter significant venous pooling in the legs.

### Implications

Prolonged uninterrupted sitting time [1] and the consumption of high concentrations of saturated fats [2] are two of the most important yet modifiable independent risk factors for CVD risk [3]. Our data suggest that when 180 min of uninterrupted sitting is combined with the consumption of a high-fat meal, cfPWV is augmented compared to that resulting from uninterrupted sitting with the consumption of a low-fat meal in a young healthy population. Given that cfPWV is an established predictor of coronary artery outcomes and morbidity [34], this finding is important. Furthermore, while there was no effect of meal type, this is the first study to show a significant decrease in the af-SG following 180 min of uninterrupted sitting. Collectively, these findings indicate that consuming a meal high in saturated fat prior to a period of uninterrupted prolonged sitting may cause additional stress on the cardiovascular system beyond sitting alone. Future studies should seek to determine (1) whether these effects are present in the female population, as well as in at-risk and elderly populations, and (2) whether the effects of novel interruption strategies such as sit-to-stand maneuvers and leg fidgeting, which have been shown to offset sitting-induced dysfunction [7], reduce this additional cardiovascular burden caused by a high-fat meal and uninterrupted prolonged sitting.

Consuming a meal high in saturated fat prior to prolonged uninterrupted sitting acutely augments known markers of CVD risk more than sitting following a low-fat meal. Future studies are needed to (1) identify the long-term effects of combining these behaviors and (2) determine appropriate interruption strategies to moderate the determinantal changes in central and peripheral cardiovascular function.
